# Rare Earth Extraction from Phosphogypsum by *Aspergillus niger* Culture Broth

**DOI:** 10.3390/molecules29061266

**Published:** 2024-03-13

**Authors:** Jiangang Zhang, Xinyue Zhang, Xiangdong Su, Haijun Du, Yongzhong Lu, Qinglian Zhang

**Affiliations:** 1 Key Laboratory of Light Metal Materials Processing Technology of Guizhou Province, Guizhou Institute of Technology, Guiyang 550025, China; zhangjg009@126.com (J.Z.); yzlu@git.edu.cn (Y.L.); 2 School of Chemical Engineering, Guizhou Minzu University, Guiyang 550025, China; 15057928600@163.com; 3 School of Chemistry and Chemical Engineering, Yili Normal University, Yining 835000, China; hjdu51@163.com; 4 School of Biology and Environmental Engineering, Guiyang University, Guiyang 550005, China; zql_emoji@163.com

**Keywords:** phosphogypsum, leaching, rare earth elements, *Aspergillus niger*, biological hydrometallurgy

## Abstract

The extraction of rare earth elements (REEs) from phosphogypsum (PG) is of great significance for the effective utilization of rare earth resources and enhancing the resource value of PG waste residues. This study used *Aspergillus niger* (*A. niger*) fungal culture filtrate as a leaching agent to investigate the behavior of extracting REEs from PG through direct and indirect contact methods. According to the ICP-MS results, direct leaching at a temperature of 30 °C, shaking speed of 150 rpm, and a solid–liquid ratio of 2:1, achieved an extraction rate of 74% for REEs, with the main elements being yttrium (Y), lanthanum (La), cerium (Ce), and neodymium (Nd). Under the same conditions, the extraction rate of REEs from phosphogypsum using an *A. niger* culture filtrate was 63.3% higher than that using the simulated organic acid-mixed solution prepared with the main organic acid components in the *A. niger* leachate. Moreover, the morphological changes observed in *A. niger* before and after leaching further suggest the direct involvement of *A. niger*’s metabolic process in the extraction of REEs. When compared to using organic acids, *A. niger* culture filtrate exhibits higher leaching efficiency for extracting REEs from PG. Additionally, using *A. niger* culture filtrate is a more environmentally friendly method with the potential for industrial-scale applications than using inorganic acids for the leaching of REEs from PG.

## 1. Introduction

Phosphogypsum (PG) is a kind of industrial solid waste produced in the manufacturing of phosphate fertilizer and phosphoric acid and is generally gray or yellow. At a global level, the massive accumulation of PG has led to significant environmental issues, the occupation of large areas, and negative impacts on water, soil, the atmosphere, and organisms [1]. According to a previous study, 4–5 tons of PG are produced for every 1 ton of phosphoric acid (calculated by 100% P_2_O_5_) [2].

China is the world’s largest producer of PG, with a current stockpile of nearly 400 million tons, which is increasing at the rate of 50 million tons/a. In the Earth’s crust, rare earth elements (REEs) mainly occur in rare earth minerals and eluvial minerals of the weathering crust [3]. Because the ionic radius of RREs (0.848–0.106 nm) is close to that of Ca^2+^ (0.106 nm), a considerable portion of REEs exist in the crystal structure of apatite in an isomorphic form [4]. Globally, the reserves of phosphate rock amount to 100 billion tons, of which about 50 million tons are associated with rare earth elements, with an average of 0.05% [5]. With the increasing shortage of rare earth resources, the trace rare earth associated with phosphate rock as a potential rare earth resource has attracted considerable attention. Approximately 90% of the world’s phosphoric acid is produced by a wet process using sulfuric acid to decompose phosphate rock; the most common dihydrate phosphoric acid process results in 70–85% of the REEs in phosphate rock being transferred to PG, whereas semi-hydrate phosphoric acid can transfer 95% of the REEs into PG [6].

It is estimated that each year, 250 million tons of phosphate ore are used in the production of phosphoric acid, and about 100,000 tons of REEs end up in PG [4]. In this sense, the recovery of rare earth from PG is a potential solution to dwindling global rare earth resources [5]. The extraction of REEs from PG generally uses inorganic acid leaching [7,8,9], applying sulfuric acid, hydrochloric acid, and nitric acid. Although the rare earth recovery rate is 80–90% and above, the acidity of PG is largely increased, resulting in higher disposal costs and environmental issues. 

Biological hydrometallurgy is generally considered an environmentally friendly alternative to traditional chemical leaching methods for the extraction of low-grade rare earth minerals, with low investment and low energy consumption [10]. Bioleaching can recover some valuable elements, using microorganisms to reduce waste toxicity. Studies have shown that the microbial leaching of rare earth resources creates favorable conditions for the migration of rare earth elements from solid into solution. This approach can save costs [11], reduce environmental pollution, and largely diminish resource waste.

The use of fungi in the bioleaching of REEs has, so far, scarcely been investigated. It has been reported that *A. niger* has been used to leach metallic elements, such as copper, zinc, uranium, manganese, and aluminum, from various substances for more than 40 years [12,13,14]. In this context, this paper used *A. niger* under different conditions and invested its leaching efficiency for rare earth elements from PG. In this process, the fungal spores, acidity, and metal complexing are supposed to be the main factors. The biomass and pH of *A. niger* in a pure culture were measured before bioleaching, and *A. niger* morphology was investigated before and after bioleaching, using a scanning electron microscope (SEM). The internal structure of *A. niger* cells and the distribution of rare earth elements before and after bioleaching were studied by transmission electron microscopy (TEM).

## 2. Results and Discussion

### 2.1. An Overview of the Phosphogypsum Sample

According to the XRF analysis, the main components of PG were oxides, with the following mass percentage concentrations (±3%): SO_3_ (50.73 wt %), CaO (37.73 wt %), SiO_2_ (4.97 wt %), P_2_O_5_ (0.91 wt %), Al_2_O_3_ (0.75 wt %), and Fe_2_O_3_ (0.5 wt %); F accounted for 3.96 wt %. The most abundant metals were Ca, Ba, Cr, Cu, Fe, K, and Mg. The gypsum (calcium sulfate) in phosphogypsum exists in the form of dihydrate calcium sulfate (CaSO_4_·2H_2_O) crystals. According to the analysis results of the ICP-MS, the following REEs (ppm) were recovered with an error of ±5%: Y (25.24 wt %), Ce (13.55 wt %), Nd (6.45 wt %), La (6.34 wt %), Gd (4.31 wt %), Sm (3.52 wt %), and Dy (3.49 wt %). The total rare earth element content of PG in the Xifeng phosphate mining area studied reached 70.04 ppm. It is the result of the low rare earth content in this phosphate rock. Due to the low content of rare earth elements, it is speculated that rare earth elements exist in a homologous form within the dihydrate calcium sulfate crystals. 

### 2.2. Pure Biological Culture

**Figure 1:**
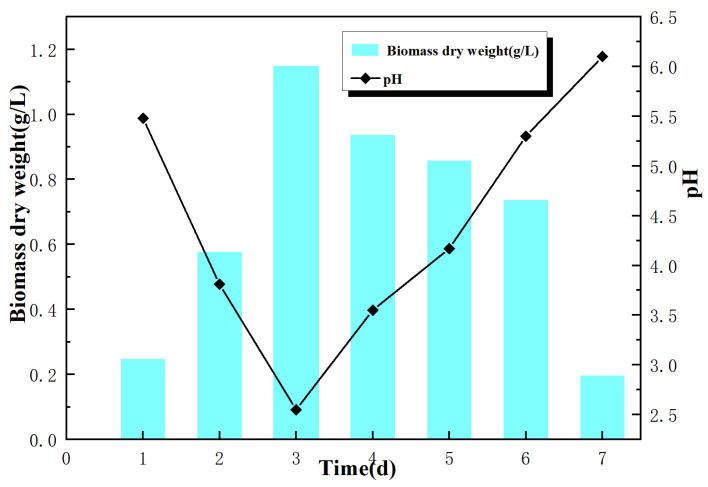
Changes in biomass concentration and pH value of *A. niger* in pure culture.

Prior to microbial leaching, the activity of *A. niger* was investigated. According to the different microbial properties and culture conditions, the growth state of *A. niger* has a certain impact on the overall process. To reduce errors, it is therefore necessary to culture pure *A. niger* under the same conditions as those of the biological leaching and to determine the fungal biomass and pH value of the culture medium within 7 days. *A. niger* spores are then dispersed in the medium on a shaking table, forming one macro agglomerated ball [15]. In previous studies, there was little or no explanation of how the *A. niger* used for metal ion leaching exerts its metabolic and morphological effects. As seen in Figure 1, the growth of *A. niger* pellets changed over time, and its biomass grew to 1.15 g/L on day 3, followed by a decrease in biomass, which may be due to the influence of metabolites produced by the microorganisms (organic acid, ferricarriers, and extracellular enzymes, among others), which, to some extent, inhibited the growth of *A. niger*. During culturing, the pH value of the medium decreased from 5.48 to 3.81 on the second day; the logarithmic growth period of *A. niger* was 2–4 days [16]. On the third day, the pH value further decreased to 2.55, when *A. niger* produced the highest amounts of organic acids [17]. After that, the pH value increased continuously, indicating the end of the active growth period of *A. niger*. The increase in the pH value in the culture medium might be the result of the lysis of cell walls and the release of intracellular metabolites and alkaline buffering properties.

### 2.3. Effects of Direct and Indirect Methods on REEs and pH Value in A. niger Leaching Process 

**Figure 2:**
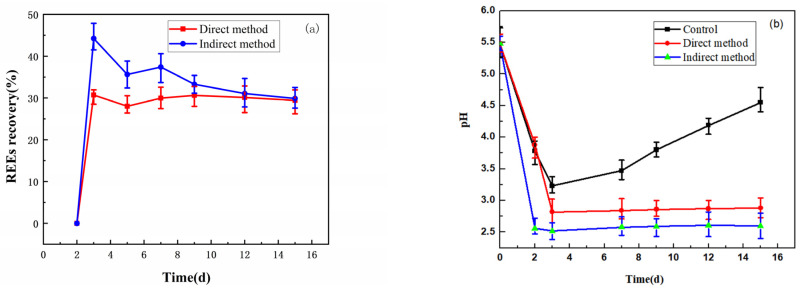
Bioleaching under different pulp concentrations: (**a**) recovery of leached REEs; (**b**) changes in the pH value of the leaching solution.

The leaching of REEs by *A. niger* by direct and indirect contact methods and their pH changes in fermentation broth were showed in Figure 2. As shown in Figure 2a, the leaching rate of rare earth elements under the two methods reached the highest value on the third day. The leaching rate of rare earth elements under the indirect contact method was as high as 44.24%, which was 13.52% higher than that under direct contact method. As can be seen from Figure 2b, there is a negative correlation between the leaching rate of rare earth elements and pH value in the whole leaching process, that is, the higher the leaching rate of rare earth elements, the lower the pH value of the fermentation liquid, which is consistent with the previous report [18]. This may be because microorganisms consume nutrients in the culture medium and produce metabolites in the growth process. Organic acids are the main reason affecting the decrease in pH value of the fermentation liquid, and low acidity can promote the chemical bond of PG to be weaker and easier to break. As shown in Figure 2b, the control experiment used pure-culture *A. niger* fermentation liquid, therefore no rare earth element leaching occurred.

**Figure 3:**
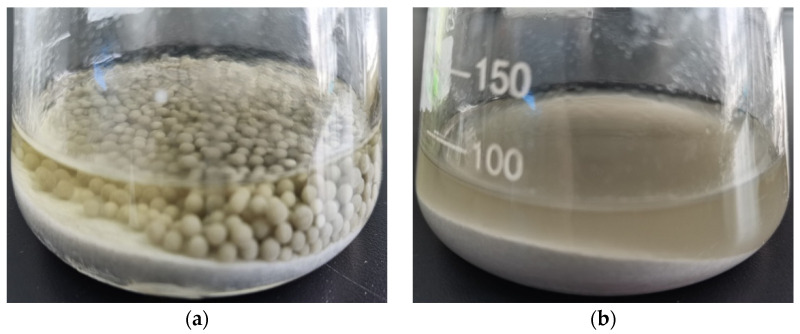
Different forms of the two methods in the leaching process: (**a**) direct method; (**b**) indirect method.

In the direct contact method, the distribution of REEs consists of two parts, the total leaching rate of the REEs in the fermentation liquid and *A. niger* strain adsorption of rare earth elements. The leached *A. niger* strain (2 g, dry weight) was ashified in a high-temperature furnace (550 °C, 3 h), and then high-grade pure nitric acid (5 mL) was added to the ashes for digestion; with a constant volume of 50 mL, the REEs were detected by ICP-MS. The results showed that the mycelia of *A. niger* were enriched by 43.69% REEs. Combined with the REEs leached into the fermentation liquid, the total leaching rate of rare earth elements was up to 74%, obviously about two times higher than that in the indirect contact method. According to previous studies, as *A. niger* strains are directly involved in leaching in the direct contact method, rare earth elements will be adsorbed to *A. niger* cells or adhere to their surfaces, forming stable complexes with carboxyl or phosphoric functional groups on the surface [19,20]. *A. niger* strains and their metabolites both play an important role in the leaching process of REEs. Figure 3 shows the different forms of the two methods in the leaching process. Therefore, the direct method is a better method for extracting trace rare earth elements from phosphogypsum with an *A. niger* culture solution.

### 2.4. Organic Acid Detection and Leaching Mechanism

The presence of organic acids was an important feature of metal leaching. The types and contents of organic acids produced by different microorganisms varied and changed with the culture conditions. Table 1 lists the different acids in the pure culture of *A. niger* in this study and the organic acid content in the triangle flask after leaching. According to these findings, *A. niger* mainly produced citrate, gluconic acid, oxalate, tartaric acid, and ketoglutaric acid during growth, with concentrations (error of ±5%) of 983.089 mg/L, 134.140 mg/L, 130.471 mg/L, 56.865 mg/L, and 32.787 mg/L, respectively.

**Figure 4:**
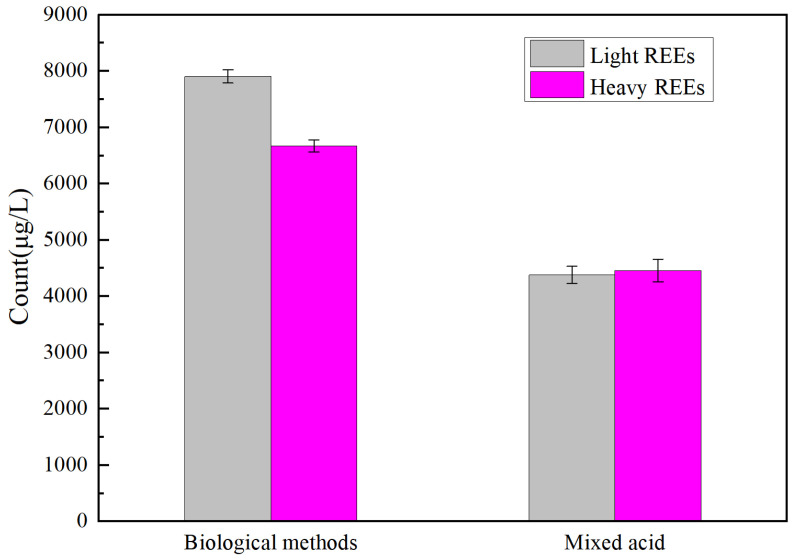
The rare earth elements in PG were extracted by mixed acid and fermentation liquid.

To prove the biological role of fungi, not just organic acids, and to determine whether the bioleaching of metal ions occurs through dissolution and chelation of organic acids, the mixed acids prepared from commercial organic acids were compared to a biological fermentation liquid. The mixed acid solution had the following composition: 1000 mg/L of citric acid, 150 mg/L of tartaric acid, 100 mg/L of ketoglutaric acid, 100 mg/L of gluconic acid, 200 mg/L of mixed acids; the bio-fermentation liquid was added at the same amount as for the PG solid samples at a solid–liquid ratio of 2:1, a temperature of 30 °C, and an oscillation rate of 150 rpm on a shaking table. The contents of rare earth elements in the supernatant of the leaching solution were determined by ICP-MS after 10 days of co-culturing. As shown in Table 2, the total amount of REEs leaching by biological method is 5735.7 μg/L more than that by organic acid method 63.3%, in which heavy REEs are increased by 33.14% and light REEs are increased by 44.59%, indicating that *A. niger* promotes the leaching of REEs from PG. As shown in Figure 4, the leaching of rare earth elements in the *A. niger* fermentation liquid was more pronounced than that of the mixed acid liquid. The rare earth elements are usually divided into two types: ceric rare earth elements, La-Eu, also known as light rare earth elements (LREEs), and yttrium rare earth elements, Gd-Lu+Y+Sc, also known as heavy rare earth elements (HREEs) [21]. In addition, in the mixed acid, the amount of heavy REEs was higher than that of light REEs, whereas in the *A. niger* fermentation liquid, the opposite pattern was observed. This indicates a certain selectivity of the *A. niger* fermentation liquid. 

### 2.5. Morphology of A. niger

#### 2.5.1. Morphological Changes in the *A. niger* Mycelium

In the microbial leaching of PG, various mechanisms were at play, such as acid solution, surface complexation, ion exchange, and redox reactions [22], resulting in the accumulation of refractory material in the acid solution [23]. Surface complexation was mainly related to the composition of the microbial surface, which was largely composed of protein polysaccharides and lipids. There were various functional groups on the surface, such as hydroxyl amino and carboxyl groups, as well as some inorganic elements, such as O, N, P, and S, which could coordinate with metal ions as coordination atoms. In *A. niger*, in the production of organic acid, siderophores also participated in the leaching process [24], and existing studies showed that iron carriers, generally divided into hydroxyl oxime acid and catechins, usually formed complexes with octahedral. During leaching, the complex coordination depended on the amounts of organic acids in solution, the stability of the chelate, and the functional groups on the microbial surface.

**Figure 5:**
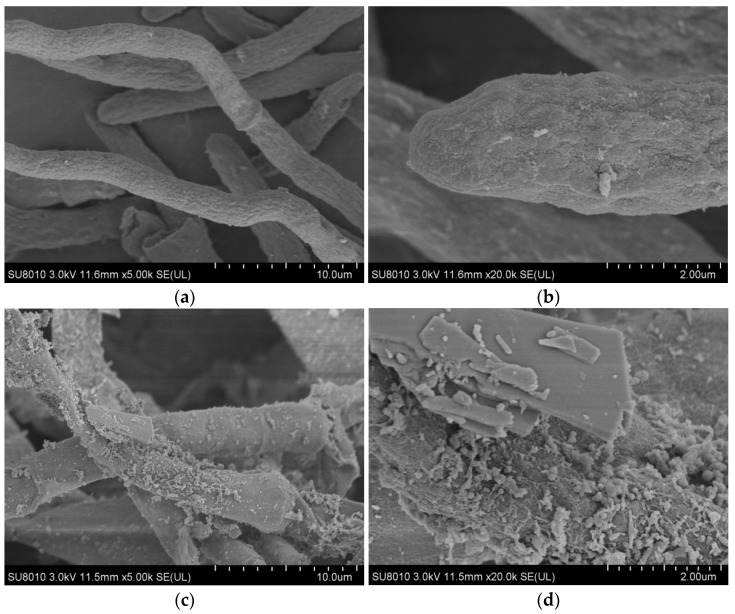
SEM images of (**a**,**b**) *A. niger* mycelium before leaching, (**c**) *A. niger* mycelium after 15 days of leaching, and (**d**) details of precipitation on *A. niger* fungal biomass after leaching.

**Figure 6:**
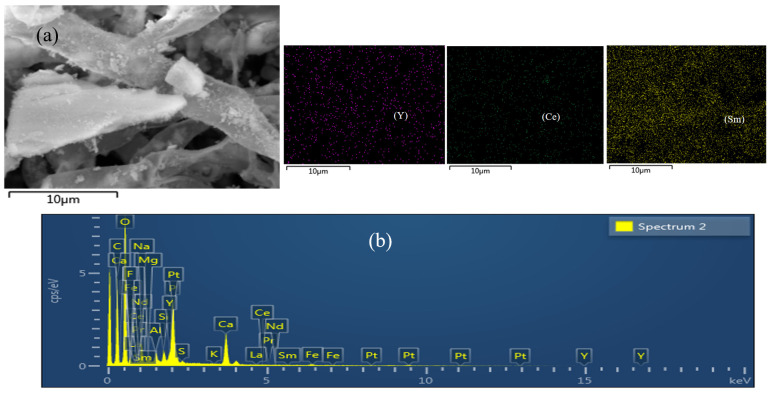
(**a**) SEM image of PG attached to *A. niger* mycelium; (**b**) EDS figure; Y, Ce, and Sm are enriched on the surface.

In order to study the morphology of *A. niger* and the distribution of REEs adsorbed before and after leaching, the morphology of *A. niger* bodies in the direct contact method was scanned. As seen in Figure 5a,b, prior to the leaching experiment, the mycelium surface was smooth. Although the shape of the mycelia was not changed after leaching (Figure 5c), granular substances could be observed on the surface. As seen in Figure 5d, the PG and *A. niger* mycelium were intertwined, and the mycelium surface was severely eroded. Figure 6 shows that a large number of REEs are enriched on the surface of *A. niger* bodies.

#### 2.5.2. Changes in *A. niger* Cells

**Figure 7:**
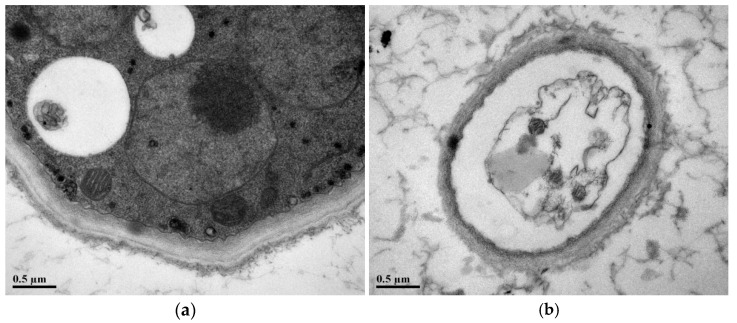
TEM images: (**a**) fungal cells before leaching; (**b**) fungal cells after leaching.

As shown in Figure 7a, before extraction, the *A. niger* cell structure was intact and clear, with an obvious cell wall, nucleus, nucleolus, mitochondria, and vacuoles, etc. As shown in Figure 7b, after extraction, although the black mold cells of *A. niger* remained intact and the cell wall was clearly visible, the organelles aggregated together. This was indicative of cell apoptosis, and similar findings had also been reported by other researchers [25]. One possible reason was that toxic substances in phosphogypsum may have damaged *A. niger* cells, leading to a decrease in cell activity and a reduction in acid production. Another possible reason was that cells maintained their metabolism by changing their morphology and specific surface area to resist the erosion of rare earth elements or heavy metals until cell apoptosis [26,27].

**Figure 8:**
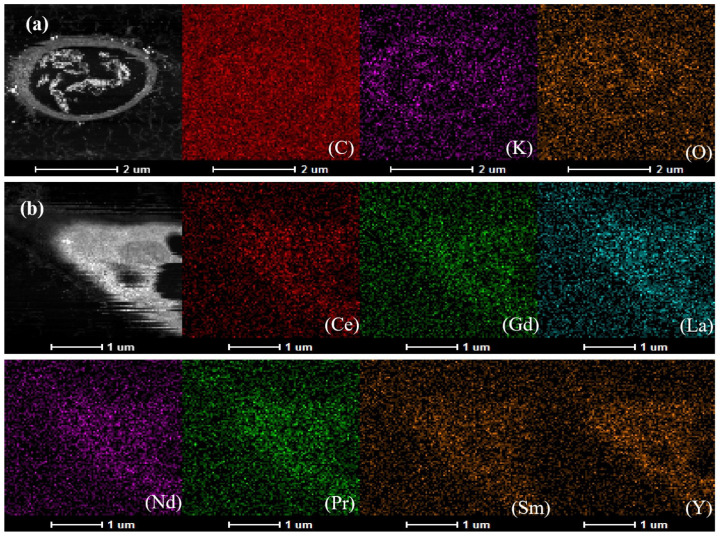
Images of *A. niger* cells and accumulated elements: (**a**) before leaching; (**b**) after leaching.

Figure 8 showed the distribution of elements in *A. niger* cells before extraction. The cells were oval-shaped. The main elements in the cells were C, K, and O, with no rare earth elements detected. After the extraction experiment, the cells underwent deformation (Figure 8b), and a large amount of Ce, Gd, La, Nd, Pr, Sm, and Y aggregated within the cells. This indicated that the rare earth elements extracted from the PG accumulated inside the cells. Both Figure 7 and Figure 8 reflected the shrinkage of *A. niger* cells after rare earth extraction, which could have served as a method for enriching rare earths using *A. niger* biomass. That is, after separating the *A. niger* biomass from the extraction liquid, drying and incinerating it would have yielded a high content of rare earth oxides. This would have avoided the need to use additional chemical reagents to precipitate and separate rare earth elements from the extraction liquid.

## 3. Materials and Methods

### 3.1. Phosphogypsum Collection

The PG samples were collected from the Xifeng phosphate mining area, China. To avoid the uneven distribution of minerals, the samples were dried and ground with an agate mortar to obtain PG particles with a particle size of less than 150 μm. In order to measure the pH value of PG, 1 g of the PG sample was weighed in a 250 mL conical flask, and 50 mL of ultrapure water was added into it, followed by shaking on a shaking table for 24 h at 250 rpm; the obtained pH was 4.55. To quantify REEs in PG, 0.5 g of PG was placed in a Teflon beaker with 2 mL of ultrapure water. Subsequently, 10 mL of high-grade pure nitric acid was added, and the mixture was slowly heated on a heating plate to evaporate the acid until it was nearly dry. After cooling slightly, 10 mL of nitric acid solution (1 + 1) was added and dissolved by heating. After cooling to room temperature, ultrapure water was used to reach a volume of 50 mL, and the mixture was filtered and set aside. For the analysis of REEs, a rare earth multi-element calibration standard solution was used to generate external standard curves, and the same method was used to prepare standard controls to evaluate the accuracy of the analysis data [28]. The concentrations of total REEs were determined by inductively coupled plasma mass spectrometry (ICP-MS, PerkinElmer, Waltham, MA, USA, NexION2000; RSD < 3%). The settings of the ICP-MS were as follows: power, 1.5 kW; plasma gas flow, 20.0 L/min; auxiliary gas flow, 2 L/min; pump speed, 35 rpm; cleaning time, 25 s. An X-ray fluorescence analyzer (XRF) was used to detect the major elements in PG.

### 3.2. Microorganisms and Growth Conditions

The Aspergillus strain was provided by Nantong Kaiheng Biotechnology Development Co., Ltd (Nantong, China). As it was in the second generation, it could be used directly to ensure strain activity. Briefly, under aseptic conditions, a sterile inoculation ring was used to transfer the fungal strain to the corresponding liquid culture medium for activation. The species *A. niger* grows at temperatures from 25 to 37 °C. The medium, prepared with 20% (*w*/*v*) potato juice, was autoclaved at 121 °C for 20 min, and the pH value was 5.4–5.5 (see Table 3 for Liquid medium component). The biomass of *A. niger* was determined according to volatile solids (VS) [9], and the organic part of the total dry weight was determined. The difference between the dry weight and the ash weight was determined as VS. The extracts were filtered through medium-speed qualitative filter paper and placed in an oven at 105 °C for 2 h. After cooling down to room temperature and weighing, the sample was combusted at 550 °C for 3 h, cooled to room temperature, and weighed again.

### 3.3. Leaching Process

The *A. niger* strains were cultured on a 20% (*w*/*v*) potato dextrose agar (PDA) slant. Spores were washed from 7-day-old cultures using a sterile solution of physiological saline (8.5 g/L NaCl). The number of spores was 108/mL, determined using a blood cell counter. A 5% spore suspension was added to 100 mL of sterilized potato liquid medium and placed in a 250 mL conical flask. There were two methods used, namely the direct contact method and the indirect contact method. In the direct contact method, *A. niger* was first cultured in potato liquid medium for 3 days, and then unsterilized PG was added. Meanwhile, in the indirect contact method, *A. niger* was first cultured in potato liquid medium for 3 days, and then filtered using medium speed qualitative filter paper to obtain fermentation broth without *A. niger* strains and body pellets. Unsterilized PG was then added to the filtrate. All cultures were incubated in a temperature-controlled oscillator at 30 °C and shaken at 150 rpm for 15 days [24]. The control experiment used pure-culture *A. niger* fermentation liquid. All experiments were performed in triplicate. Briefly, 2 mL of leaching solution was taken out at regular intervals to determine the acidity and various REE contents of the fermentation liquid. Simultaneously, the same amount of sterile normal saline was added. For solid–liquid separation, the mixture was centrifuged (4000 rpm, 10 min), and the supernatant was collected and diluted 100 times.

The concentration of REEs in the solution was detected via ICP-MS, and the recovery rate was calculated. The pH was measured using a glass pH electrode (PHS-3C). *A. niger* mycelia were gold-plated with palladium for 4–5 min in a Hitachi E-1010 ion sputter coater and then observed under a scanning electron microscope (SEM) (Hitachi SU-8010, Hitachi, Tokyo, Japan). The cell morphology of *A. niger* before and after leaching was characterized using a transmission electron microscope (TEM) (Hitachi H-7650).

## 4. Conclusions

In this study, *A. niger* was used as the research object to extract REEs from PG, with the following findings:

(1) The fungus *A. niger* exists widely in nature and is easy to culture. Although glucose is used as the substrate for *A. niger* culture in this study, cheap organic matter can also be used for culture to reduce the output cost.
(2) In the direct contact method, the maximum leaching rate under the optimum leaching condition is 74%, The amount of leached light rare earth elements was higher than that of heavy rare earth elements.
(3) Several organic acids were found in the *A. niger* fermentation broth, such as citric acid, gluconic acid, oxalic acid, etc., which achieved the leaching of rare earth elements through dissolution and chelation. The metabolic process of *A. niger* cells significantly improved the leaching rate of rare earth elements.
(4) Based on the SEM-EDS and TEM-EDS results, the surface and lumen of the *A. niger* cells contained large amounts of rare earth elements after leaching.

REEs leaching using *A. niger* is an environmentally friendly approach and a viable alternative to the use of mixed acid solutions. This approach can be used in the large-scale extraction of rare earth elements from PG and, perhaps, other waste products.

## Figures and Tables

**Table 1:** Changes in organic acids before and after leaching (error of ±5%).

Organic Acids	Citric Acid	Glucose Acid	Oxalic Acid	Tartaric Acid	Ketone Glutaric Acid
Before (mg/L)	983.089	134.14	130.471	56.865	32.787
After (mg/L)	4.665	167.868	72.138	63.331	33.988

**Table 2:** The concentration of rare earth elements leached by bioleaching and organic acid leaching methods (error of ±5%).

	Light REEs/μg/L										
Element symbol	La 139	Ce 140	Pr 141	Nd 142	Sm 152	Eu 153					∑REES
Biological method	1501.18	3289.46	387.46	2060.52	539.78	126.58					7905.01
Organic acid method	831.77	1822.62	214.69	1141.69	299.08	70.15					4380.02
	Heavy REEs/μg/L										
Element symbol	Sc 45	Y 89	Gd 158	Tb 159	Dy 164	Ho 165	Er 166	Tm 169	Yb 174	Lu 175	∑REES
Biological method	0.00	4843.04	712.03	66.57	461.41	85.08	288.90	28.81	167.28	17.42	6670.50
Organic acid method	0.00	3237.98	476.05	44.51	308.49	56.88	193.15	19.26	111.84	11.66	4459.81

**Table 3:** Composition of the different growth media. PDA is potato dextrose agar.

Medium Component	Potato Liquid Medium	PDA Medium
Glucose	20 g/L	20 g/L
MgSO_4_·7H_2_O	1.5 g/L	1.5 g/L
KH_2_PO_4_	3 g/L	3 g/L
Vitamin B1 trace	8 mg/L	8 mg/L
Agar	0 mg/L ^a^	20 mg/L

^a^ the liquid medium contained no agar.

## Data Availability

The data that support the findings of this study are available on request from the corresponding author. The data are not publicly available due to privacy or ethical restrictions.
